# Parents or Peers? Predictors of Prosocial Behavior and Aggression: A Longitudinal Study

**DOI:** 10.3389/fpsyg.2019.02379

**Published:** 2019-10-22

**Authors:** Elisabeth Malonda, Anna Llorca, Belen Mesurado, Paula Samper, M. Vicenta Mestre

**Affiliations:** ^1^Basics Psychology, University of Valencia, Valencia, Spain; ^2^National Scientific and Technical Research Council (CONICET), Buenos Aires, Argentina

**Keywords:** parenting styles, peer attachment, aggression, prosocial behavior, adolescence, longitudinal studies

## Abstract

The aim of this longitudinal study was to determine the associations among peer attachment, warmth from the mother and father, strict control by the mother and father, prosocial behavior, and physical and verbal aggression in adolescence. Few longitudinal studies have examined how peer attachment and parenting styles of the mother and father relate to prosocial behavior and aggression. Participants were 192 boys and 255 girls (*M* = 14.70 years; *SD* = 0.68) in wave 1. In the study participated 11 schools. For three successive years, participants reported on their fathers’ and mothers’ warmth and strict control, peer attachment, prosocial behavior, and aggression. Structural equations modeling was employed to explore two longitudinal models. Results show the influence of the mother and father on prosocial and aggression during adolescence. In addition, strong peer attachment predicted prosocial behavior in subsequent years. Therefore, the findings indicate that despite the increasingly important role of friends during the transition from childhood to adolescence, parenting styles play a key role in the personal and social development of their children. Programs aimed at preventing aggression should be designed considering the importance of stimulating and strengthening prosocial behavior, peer attachment and a family environment of affect, support and communication.

## Introduction

Prosocial behavior and aggression are commonly observed social behavior that have important health and social implications (Carlo, 2006; Carlo et al., 2012a). Furthermore, studies show that they are two closely related constructs: individuals who have low levels of empathy and prosocial behavior, tend to have low tolerance of others and also tend to be highly aggressive (Nelson and Baumgarte, 2004; Samper et al., 2008; Carlo et al., 2010a).

Prosocial behavior is voluntary behavior aimed at benefiting others. Such behavior includes sharing, caring and helping (Batson, 2011). Recent years have witnessed an increase in research on prosocial behavior. Scholars have reported the benefits of prosocial behavior in social relationships, citing prosocial behavior as a facilitator of people’s adjustment from childhood to adulthood (Caprara, 2014; Luengo et al., 2014) and as a protective factor in reducing the risk of aggressive and problematic behavior (Tur-Porcar et al., 2004; Van der Graaff et al., 2012; Caprara, 2014; Mestre, 2014). Prosocial behavior enables social relationships and mitigates undesirable behavior while encouraging well-being and social adjustment, which in turn lead to positive development. The ability to detect and respond to requests for help and support not only protects individuals throughout their development, but also encourages sympathy, gratitude and appreciation. In other words, prosocial tendencies promote popularity among peers and help people to trust their own skills and positive emotions (Caprara et al., 2012; Caprara, 2014; Luengo et al., 2014).

In contrast, human aggression is considered as ‘any behavior directed toward another individual that is carried out with the immediate intent to cause harm. In addition, the perpetrator must believe that the behavior will harm the target, and that the target is motivated to avoid the behavior’ (Anderson and Bushman, 2002, p.28). Aggressive behavior hampers social relationships and seriously affect victims. The study of aggressive behavior has awakened widespread interest among psychology scholars because of the effects of aggressive behavior on both perpetrators and victims (Card et al., 2008). According to positive psychology, prosocial behavior is the antithesis of aggressive behavior because of both the consequences of interactions with others and the benefits or negative consequences for oneself. Empathy and prosocial behavior are the most notable factors that are capable of protecting adolescents from developing aggressive and maladaptive behavior (Van der Graaff et al., 2012).

Parenting styles refer to the emotional atmospheres shaped by parental attitudes during the process of children socialization (Darling and Steinberg, 1993). Two important dimensions of parenting styles, warmth (or support) and control have been fully studied by developmental scholars (Maccoby and Martin, 1983; Baumrind, 1991; Carlo et al., 2010b). The quality of parenting styles vary according to differences in the levels of parental warmth and affection, and parental control, in reference to the granting of autonomy, and both of these constructs are associated with child and adolescent development and well-being (Broderick and Blewitt, 2003; Llorca et al., 2017b).

As Carlo et al. (2012a) postulate, attachment and social support theories are developmental approaches that hypothesize positive social behavioral outcomes for individuals who build and maintain secure attachments to parents and peers (Carlo et al., 2012a).

According to socialization theorists (Bandura, 1986; Hoffman, 2000), parents play a key role in promoting social adjustment and prosocial behavior in their children. In general, children *psychosocial adjustment* is greater in families that engage in affective and communicative behavior and establish rules governing parent-child relationships. Studies show that parental warmth and support have been positively associated with children prosocial behavior (Yarmurly and Sanson, 2009; Eisenberg et al., 2015; Llorca et al., 2017b). Additionally, parental control combined with strict verbal and physical disciplining practices could lead to aggressive and antisocial behavior (Maccoby and Martin, 1983; Hoffman, 2000; Eisenberg and Valiente, 2002). An excess of parental control may deprive children and adolescents of the chance to develop their autonomy. On the contrary, parents’ warmth and affection—characterized by positive endorsement of the child—coupled with a set of coherent, consistent rules encourages children psychological adjustment (Carlo et al., 2010b; Khaleque, 2013). In this study, we have focused on these two dimensions: warmth and strict control.

Likewise, Attachment Theory (1988, 1995) offers a frame in which this lattice work of variables makes sense. Attachment is defined by Bowlby (1988, 1995) as the stable affective bond that people have with their significant others, mainly with parents, from their first years of life. The affective bond, in this sense, relates to a profound need to maintain contact and closeness with a carer, but also with the quality of the interactions. This means that the development of the affective bond is subject to the quality of the relationships established between the child and their main caregivers (Carrillo, 2008), where the parenting styles developed by the father and the mother come into play. Therefore, the way in which the parenting style is made up can be considered as an important predictor of the kind of human being developed (Duarte-Rico et al., 2016). Bowlby (1988, 1995) proposed that an attachment behavior system regulates the responses of the child and the adolescent when they experience emotional anguish. Also that the attachment figures who offer contact, calm and comfort facilitate the development of the emotional regulation and well-being of the child and the adolescent, as well as the expectations that close relations will provide a safe haven and a secure base. These conditions, in turn, are thought to stimulate the development of positive models of self and others in relationships, benefiting peer attachment.

Parental warmth can be defined as the presence of positive affect, responsiveness, support and good communication in parent-child relationships, and encouragement of autonomy of the children based on discipline (Baumrind, 1991). In this sense, parenting styles based on support instill in children and adolescents the limits of socially adapted behavior, and this is reflected in higher moral competency in children (Hoffman, 2000). On the other hand, control is an important feature of parenting styles and is related to both the authoritative and authoritarian styles (Baumrind, 1991). Two types of control have been recognized in the literature, behavioral control and psychological control (Barber, 2002; Bugental and Grusec, 2006). Behavioral control includes parental behavior used to manage the daily activities of their and monitor some aspects of their lives (Barber, 1996), whereas psychological control is defined as systematic parental use of manipulative techniques, such as the instigation of guilt and withdrawal of love (Barber, 1996). In contrast, parental strict control refers to the degree of strictness, behavioral rules, and negative evaluations and expectations imposed upon children by parents. Several studies have consistently shown that parental warmth from both the father and mother is positively associated with sympathy, prosocial moral reasoning and prosocial behavior. Furthermore, these studies have shown that parental warmth is a more reliable predictor of prosocial development than parental strict control (Carlo et al., 2010b; Padilla-Walker et al., 2011; Kuppens et al., 2013; Mestre, 2014).

A longitudinal study during the latter years of childhood and early years of adolescence (9-14 years old) in Spain showed that parenting styles characterized by warmth - especially from the mother -, communication, trust, control over rules and obeying rules combined with empathy and prosocial moral reasoning are predictors of prosocial behavior in children. Conversely, excessively rigid strict control by parents is negatively related to prosocial behavior (Carlo et al., 2010b).

Parenting styles of the father and mother also predict aggressive behavior in children. Aggression in children and adolescents have been associated with negative paternal practices characterized by an authoritarian style with regular punishments, excessive criticism and extremely rigid or permissive attitudes (Kuppens et al., 2013; Calvete et al., 2014). Guided by social learning theory (Bandura, 1986; Hoffman, 2000), a hostile form of parenting that offers little emotional support, displays little tolerance of children’s behavior and imposes strict discipline during childhood predicts hostility and rage during adolescence (Hakulinen et al., 2013). Similarly, adolescents who perceive in their relationships with their father and mother strict control characterized by negative evaluation and excessively rigid attitudes are less emotionally stable and have greater trouble controlling their impulses and regulating their emotions (Samper et al., 2015). In contrast, authoritative parenting and associated dimensions, such as warmth, positive affect, behavioral control, responsiveness, and autonomy-support, have been inversely related to aggression (Brown et al., 2007; Leadbeater et al., 2008) among children and adolescents (Clark et al., 2015). Spain is considered a society that values family, in fact, the majority of the general population and young Spaniards value family as the most important institution (CIS, 2014: Centro de Investigaciones Sociológicas, the Spanish Centre for Sociological Research; INJUVE, 2012: Spanish National Youth Institute).

Attempts to determine whether the parenting style of the mother or of the father has a greater influence on the behavior of children have been inconclusive (Ruiz-Hernández et al., 2018). Whereas some researchers have reported that children aggressiveness is more closely related to factors associated with the mother’s parenting, characterized by strict control, negative evaluation of the child and excessive criticism (Tur-Porcar et al., 2012), others have shown the importance of relationships with both parents (Hoeve et al., 2011; Yoo et al., 2013; Gómez-Ortiz et al., 2015). Fathers are increasingly involved in their children’s education, acquiring an important parenting role (Ruiz-Hernández et al., 2018).

In this sense, the researchers have analyzed together as a whole the parenting styles of both parents. There have been only a few studies (Tur-Porcar et al., 2012; Wahl and Metzner, 2012; Sun et al., 2017) in which parenting styles from both parents and their effects on children emotional development and behavior have been separately analyzed. In these studies, maternal resources (among which is warmth consistency from childhood through adolescence) appeared to have a cumulative impact on daughters, but the process for sons was compensatory. Differences in the established roles of the father and of the mother in predicting children behavior and emotional development imply that gender is an valuable factor in research on parenting style (Wood and Eagly, 2012). For this reason, in this study, we kept separate the models with fathers and mothers.

Adolescence is a transitional stage during which relationships with peers gradually become more and more important. In fact, during adolescence, peer attachment provide a secure haven, unlike during childhood, when needs for affect are satisfied mainly by parents (Zeifman and Hazan, 2008). Adolescence is thus a period of transition from dependence on parental relationships to close relationships with peers. Between the beginning of adolescence and early adulthood, peer attachment grows significantly (Delgado et al., 2011). In this period, the peer group occupies a main role due to the attempt of the adolescent to gain more autonomy and differentiation from the parents, who will move to a secondary role, further developing their own identity as well as the expansion of affective relationships (Penagos et al., 2006). Close relationships with peers and a positive family environment can act as protective factors against aggression and can even reduce the effects of low empathy as an enhancer of behavior intended to harm others (Batanova and Loukas, 2014).

Managing peer relationships is one of the most challenging and significant developmental tasks adolescents face (Allen and Loeb, 2015). Building solid relationships with peers appears crucial to healthy social development in adolescence (Allen and Loeb, 2015; Llorca et al., 2017a). In this sense, although the findings reported in research have noted that peers might be a source of negative influence on adolescents and adults (Gardner and Steinberg, 2005; Dishion and Tipsord, 2011), others have asserted that peers could also serve as resources for fostering prosocial development (Van Hoorn et al., 2014). These scholars have pointed out that the relationship with peers makes possible tasks like the establishing of positive and reciprocal social relations, regulation and control of aggression, the increase of social competence and prosocial development. All these achieved through reinforcement, punishment or the imitation of peers (Guijo, 2002; Carlo, 2006).

As we noted earlier, the main caregivers are essential for socialization. Later, peers provide security and emotional support (Escobar et al., 2011), all of which contributes to greater psychological adjustment. In a study of Spanish adolescents aged 13–14 years, Samper et al. (2015) concluded that parenting styles with high levels of affect and communication were related to empathy, which in turn was related to peer attachment. The same study showed that, conversely, strict control by the father and mother predicted emotional instability, although this instability did not have a direct relationship with peer attachment. In another study into relationships between parenting and peer attachment, adolescents who recalled high parental affect reported higher scores in peer attachment than adolescents who recalled low parental affect. The mother’s influence on peer attachment was higher than the father’s (Delgado et al., 2011).

Although studies have shown the important role of parenting styles in the behavior of children and adolescents, few studies have adopted a longitudinal design to show the effect of parenting styles, warmth and strict control, and peer attachment on the adjustment of adolescents, prosocial behavior and aggression, in the same model (Carlo et al., 2010b; Batanova and Loukas, 2014; Calvete et al., 2014).

In longitudinal studies, the results about prosocial and aggressive behavior and peer attachment have revealed that gender differences increase with age until later adolescence (Garaigordobil and García de Galdeano, 2006; Van der Graaff et al., 2012). During childhood, parents usually satisfy the attachment needs of their children. Scholars have shown the effect of parent-child relationships as the antecedent of relationships among peers, with gender as a mediator in interpersonal relationships (Cai et al., 2013; Pérez and Alvarado, 2015). Nevertheless, relationships with peers become increasingly important for adolescents as these relationships begin to satisfy their attachment needs, with girls scoring higher than boys for peer attachment (Laible et al., 2000; Moretti and Peled, 2004). These gender differences diminish in the period of later adolescence. In this sense, adolescence marks a period of transition due to the attempt of the adolescent to gain more autonomy and differentiation from the parents (Allen et al., 2017).

The present study examined the effect of the mother and father’s parenting styles - in terms of warmth and strict control - and peer relationships in terms of attachment on prosocial behavior and physical and verbal aggression in adolescence. The main aim of this longitudinal study was to determine the relationship among peer attachment, warmth from the mother and father, strict control by the mother and father, prosocial behavior, and aggression in adolescence. To better understand how parenting styles and peer attachment might be related to both prosocial and aggressive behavior, two models were examined in this study. Although mediating relations are possible, some researchers have reported and found evidence that parenting and peers might interact to predict outcomes (Allen et al., 2017; Llorca et al., 2017a). The first model assessed relationships among the mother’s parenting style, peer attachment and prosocial and aggressive behavior over time. The second model assessed the relationships among the father’s parenting style, peer attachment and prosocial and aggressive behavior over time. Potentially, parenting styles and peer attachment are directly related to prosocial behavior and aggression. Examining these two models gave us a clear understanding of how warmth, strict control and peer attachment are associated with both positive behavior and aggressive behavior. The relationship between peer attachment and parenting styles and their influence on prosocial and aggressive behavior were tested at different moments throughout adolescence. The findings respond to the following question regarding the relative importance of parenting and peers: Are parenting styles important during adolescence, or does peer attachment take over their role?

In this study, we expected that parental warmth would be positively related with peer attachment, whereas we expected strict control by parents to be negatively related to peer attachment. We also hypothesized that peer attachment would be positively related to prosocial behavior and negatively linked to aggression. Furthermore, parental warmth would be positively related to prosocial behavior and negatively related to aggression. In contrast, strict control by parents to be negatively associated with aggression. Finally, peer attachment and parenting styles would be related to prosocial behavior and physical and verbal aggression throughout adolescence.

## Materials and Methods

### Participants

Five hundred Spanish adolescents participated in a three-wave longitudinal study in Valencia, Spain. However, only four hundred and seventeen adolescents fully completed all three surveys. Therefore, the final sample consisted of 192 boys and 225 girls. In the first wave, adolescents were either in the third year of compulsory secondary school (81 boys and 85 girls) or the fourth year of compulsory secondary school (111 boys and 140 girls). Furthermore, additional analyses were reported. We have done *T*-test Analysis to assess whether the means of both groups (students who participate in the first wave and students who were present in all three waves of data collection) were statistically different from each other. We ascertained that there were no differences in the major variables between both groups for all three waves of data collection. In total, 11 schools participated in the study. Participating schools were randomly selected from both semi-private (40.3%) and public schools (59.7%). The mean age was 14.70 (*SD* = 0.68; range = 13-17 years). This study monitored participating adolescents for 3 years (see Table 1).

**TABLE 1:** Descriptive statistics.

	**Wave 1**		**Wave 2**		**Wave 3**	
	***n***	***%***	***n***	***%***	***n***	***%***
**Level of studies**						
3d Compulsory secondary school	166	39.8	6	1.4	−	−
4th Compulsory secondary school	251	60.2	164	39.3	24	5.8
1st Upper secondary school	−	−	241	57.8	170	40.7
2d Upper secondary school	−	−	−	−	214	51.3
Vocational training	−	−	6	1.4	9	2.2
**Age**						
13	14	3.4	−	−	−	−
14	135	32.4	7	1.7	−	−
15	232	55.6	125	30.0	14	3.4
16	34	8.2	237	56.8	136	32.6
17	2	0.5	44	10.6	230	55.2
18	−	−	4	0.9	35	8.4
19	−	−	−		2	0.5

In most cases, adolescents came from two-parent households where parents were married (83.7% married; 13.2% divorced). In relation to educational attainment, most mothers had a secondary school diploma or equivalent (42.2%), 30.7% had some university education, and 21.8% of mothers had less than a secondary school diploma. Likewise, 41% of fathers had a high school diploma or equivalent, 28.7% had some university education, and 24% had less than a high school diploma. Most participants self-identified themselves as being from Spain (86.6%). Small percentages of the remaining students self-identified themselves as being from Latin America (e.g., 3.4% from Ecuador, 2% from Colombia and 1.1% from Bolivia) and Eastern European countries (e.g., 1.7% from Romania). Participating students were randomly selected from the list of all schools in Valencia with students enrolled in Compulsory Secondary Education. In total, 11 schools participated in the study.

### Procedure

The questionnaires were administered by trained examiners in the classroom in 50-min sessions during school hours. The annual assessments took place in three consecutive years during the first term of the school year. The study was introduced to the teachers of the schools. Furthermore, the authorization of the Valencian Government was obtained and written informed consent was obtained from the parents of the adolescents under the age of 16. Their participation was voluntary and anonymous, taking into consideration all the ethical principles pertaining to studies carried out on human beings included in the Helsinki Declaration, under current regulations. The scientific research was reviewed and approved by the Ethics Committee of the University of Valencia. In the first wave, adolescents were identified with a code from their name, surname, level and educational center, which they maintained in the two subsequent waves.

### Measures

*Child’s Report of Parental Behavior Inventory* (CRPBI) (Schaefer, 1965; Spanish adaptation by Samper et al., 2006). This instrument assesses the child’s perceptions of family discipline in relationships with the child’s mother and father. The order of CRPBI-mother and CRPBI-father administration was counterbalanced. Example item is, ‘He (she) likes talking to me’. Participants specified their agreement with each statement using a three-point scale (*completely agree*, *sometimes*, *completely disagree*). Students responded once thinking of their father and once thinking of their mother. For this study, we selected two factors from the instrument: support and communication, and strict control. The factor *support and communication* (19 items) describes relationships based on feelings of emotional support from the father and mother, the sending of messages of affect and support, encouragement of autonomy based on discipline, and good communication between parents and children. The factor *strict control* (12 items) describes relationships based on strict control, irritability, and negative evaluation and rejection of the child. The scales had acceptable indices of reliability for all three evaluations (T1, T2 and T3, respectively - *support and communication* mother: alpha = 0.88; 0.90; 0.91 and father alpha = 0.89; 0.90; 0.92; *strict control* mother alpha = 0.80; 0.76; 0.79 and father alpha = 0.78; 0.80; 0.78). The fit statistics of the questionnaire where appropriate for the mother T1 (*support*): χ^2^ = 27.09/25, *p* = 0.35, CFI = 0.99 and SRMR = 0.02; T1 (*strict control*): χ^2^ = 90.59/51, *p* = 0.000, CFI = 0.94 and SRMR = 0.03; T2 (*support*): χ^2^ = 35.21/24, *p* = 0.06, CFI = 0.98 and SRMR = 0.02; T2 (*strict control*): χ^2^ = 78.14/48, *p* = 0.003, CFI = 0.96 and SRMR = 0.04; T3 (*support*): χ^2^ = 44.88/39, *p* = 0.23, CFI = 0.99 and SRMR = 0.02; T3 (*strict control*): χ^2^ = 112.70/51, *p* = 0.000, CFI = 0.92 and SRMR = 0.05, and, for the father T1 (*support*): χ^2^ = 25.04/23, *p* = 0.34, CFI = 0.99 and SRMR = 0.02; T1 (*strict control*): χ^2^ = 56.67/51, *p* = 0.27, CFI = 0.99 and SRMR = 0.03; T2 (*support*): χ^2^ = 25.88/23, *p* = 0.30, CFI = 0.99 and SRMR = 0.02; T2 (*strict control*): χ^2^ = 131.45/70, *p* = 0.000, CFI = 0.95 and SRMR = 0.04; T3 (*support*): χ^2^ = 72.98/41, *p* = 0.001, CFI = 0.98 and SRMR = 0.03; T3 (*strict control*): χ^2^ = 78.92/50, *p* = 0.005, CFI = 0.96 and SRMR = 0.04.

*Peer Attachment* (from the IPPA, *Inventory of Parent and Peer Attachment*) (Armsden and Greenberg, 1987). The IPPA items were translated from English to Spanish by expert researchers in the subject and later, said translation was revised by an approved translation expert. This 12-item instrument evaluates behavioral and affective/cognitive dimensions, communication, trust, and alienation, related to peer attachment. For this study, we only took the measure of attachment to peers but not to parents. Example item is, ‘My friends respect my feelings’. Cronbach’s alpha for this study was 0.75 at T1, 0.83 at T2 and 0.84 at T3. The fit statistics of the questionnaire where appropriate (T1: χ^2^ = 30.67/17, *p* = 0.02, CFI = 0.98 and SRMR = 0.02; T2: χ^2^ = 32.03/19, *p* = 0.03, CFI = 0.99 and SRMR = 0.02; T3: χ^2^ = 281.79/176, *p* = 0.000, CFI = 0.96 and SRMR = 0.03).

*Prosocial Behavior Scale* (Caprara and Pastorelli, 1993; Spanish adaptation by Del Barrio et al., 2001). This instrument uses 15 items to evaluate the behavior of help, in a unidimensional scale. Respondents indicate the frequency with which the behavior in each statement occurs (*often*, *sometimes*, *never*). Example item is, ‘I help my peers to do their homework’. Cronbach’s alpha for this study was 0.75 at T1, 0.75 at T2 and 0.74 at T3. The fit statistics of the questionnaire where appropriate (T1: χ^2^ = 75.57/33, *p* = 0.000, CFI = 0.91 and SRMR = 0.04; T2: χ^2^ = 46.24/30, *p* = 0.02, CFI = 0.97 and SRMR = 0.04; T3: χ^2^ = 50.49/29, *p* = 0.008, CFI = 0.96 and SRMR = 0.03).

*Physical and Verbal Aggression Scale* (Caprara and Pastorelli, 1993; Spanish adaptation by Del Barrio et al., 2001). This questionnaire uses 20 items to assess behavior that harm others physically or verbally, in a unidimensional scale. Respondents indicate the frequency with which the behavior in each statement occurs (*often*, *sometimes*, *never*). Example item is, ‘I threaten others.’ Cronbach’s alpha for this study was 0.81 at T1, 0.82 at T2 and 0.83 at T3. The fit statistics of the questionnaire where appropriate (T1: χ^2^ = 147.87/85, *p* = 0.000, CFI = 0.92 and SRMR = 0.05; T2: χ^2^ = 120.39/76, *p* = 0.000, CFI = 0.95 and SRMR = 0.05; T3: χ^2^ = 168.70/83, *p* = 0.000, CFI = 0.91 and SRMR = 0.06).

### Statistical Procedure

Firstly, SPSS 19 was used to estimate means and standard deviations and to calculate repeated measures analysis of variance (ANOVA) to test for mean differences across time and genders, men and women. Secondly, correlation analysis was conducted to test the relationships among the studied variables. Lastly, structural equations modeling (SEM) in Mplus 6.1 (Muthén and Muthén, 2010) was used to explore two longitudinal models. The following goodness-of-fit indices were used: chi-square divided by degrees of freedom (χ^2^/d.f.), and Bentler Comparative Fit Index (CFI). Standardized Root Mean Residual (SRMR) was used to measure error. Indirect effects were tested using the bias corrected bootstrap confidence interval method in *Mplus* (Williams and MacKinnon, 2008; Lau and Cheung, 2012). Additionally, and in order to compare the models, the Akaike’s information Criterion (AIC) and Bayesian Information Criterion (BIC) were estimated. Models with the lowest AIC and BIC are preferred optimal (Akaike, 1987).

## Results

### Preliminary Analyses and Descriptive Statistics

Table 2 presents means, standard deviations and results for the repeated measures analysis of variance (ANOVA) testing mean differences across the waves. Aggression decreased significantly between waves 1 and 2 (Bonferroni = 0.043, *p* < 0.001) and between waves 1 and 3 (Bonferroni = 0.055, *p* < 0.001). No significant differences were found for prosocial behavior. Prosociality levels remained constant over time.

**TABLE 2:** Descriptive statistics and repeated measures ANOVAS.

	**Wave 1**		**Wave 2**		**Wave 3**		***F*Test**
	***M***	***SD***	***M***	***SD***	***M***	***SD***	***F* (2, 415)**
Prosocial behavior	2.54	0.27	2.55	0.29	2.55	0.29	0.29
Aggression	1.34_1,2_	0.24	1.30_1_	0.25	1.28_2_	0.25	10.69^∗∗∗^

*^∗∗∗^*p* < 0.001. _1_Significant differences between wave 1 and wave 2. _2_Significant differences between wave 1 and wave 3.*

Repeated measures analysis of variance testing mean differences by gender across the waves were examined. Girls reported higher scores than boys in mother warmth (wave 2 and 3) and father warmth (w3), peer attachment (wave 1, 2 and 3) and prosocial behavior (wave 1, 2 and 3) but lower scores in the father’s strict control (wave 1, 2 and 3) and aggression (wave 1, 2 and 3). Furthermore, for girls, father warmth increased significantly between waves 1 and 3, and father strict control (between wave 1 and 3) and aggression decreased significantly between waves 1 and 2 and waves 1 and 3. For boys, peer attachment and aggression decreased between wave 1 and wave 2. No significant differences were found for the other variables (see Table 3).

**TABLE 3:** ANOVAS with repeated measures for waves 1, 2, and 3 by gender.

		**Wave 1**		**Wave 2**		**Wave 3**		
		***M***	***SD***	***M***	***SD***	***M***	***SD***	***F* (1, 415)**
Mother warmth	Boys	2.18	0.37	2.17	0.40	2.19	0.44	0.18
	Girls	2.25	0.40	2.27	0.42	2.31	0.47	2.59
	*F* (1, 400)	3.64		5.09^∗^		6.50^∗^		
Mother strict control	Boys	1.84	0.38	1.79	0.36	1.77	0.38	2.75
	Girls	1.77	0.35	1.72	0.35	1.71	0.37	2.65
	*F* (1, 399)	3.34		3.54		2.34		
Father warmth	Boys	2.07	0.42	2.07	0.45	2.08	0.47	0.22
	Girls	2.11	0.40	2.15	0.41	2.19	0.46	3.77^∗^
	*F* (1, 368)	1.21		3.49		4.54^∗^		
Father strict control	Boys	1.78	0.37	1.79	0.38	1.73	0.36	2.74
	Girls	1.70	0.36	1.66	0.37	1.63	0.36	3.50^∗^
	*F* (1, 367)	3.88^∗^		10.86^∗∗^		7.09^∗∗^		
Peer attachment	Boys	3.60	0.49	3.51	0.52	3.52	0.50	2.99^∗^
	Girls	3.92	0.49	3.88	0.54	3.92	0.58	0.99
	*F* (1, 415)	44.98^∗∗∗^		48.91^∗∗∗^		52.55^∗∗∗^		
Prosocial behavior	Boys	2.47	0.29	2.47	0.30	2.48	0.29	0.46
	Girls	2.60	0.23	2.61	0.26	2.60	0.27	0.37
	*F* (1, 415)	27.39^∗∗∗^		26.89^∗∗∗^		17.03^∗∗∗^		
Aggression	Boys	1.41	0.28	1.37	0.27	1.36	0.29	5.00^∗∗^
	Girls	1.28	0.21	1.24	0.20	1.22	0.19	6.42^∗∗^
	*F* (1, 415)	29.89^∗∗∗^		29.51^∗∗∗^		31.72^∗∗∗^		

*^∗^*p* < 0.05; ^∗∗^*p* < 0.01; ^∗∗∗^*p* < 0.001.*

The correlations showed that the mother’s and father’s warmth and peer attachment were all positively related to prosocial behavior and negatively related to aggression (waves 1, 2 and 3), whereas the mother’s strict control and the father’s strict control were positively related to aggression (waves 1, 2 and 3) (see Table 4).

**TABLE 4:** Correlations among the variables at waves 1, 2, and 3.

	**1**	**2**	**3**	**4**	**5**	**6**	**7**	**8**	**9**	**10**	**11**
1. Mother warmth (T1)	–										
2. Mother strict control (T1)	–0.27^∗∗^	–									
3. Father warmth (T1)	0.65^∗∗∗^	–0.22^∗∗∗^	–								
4. Father strict control (T1)	–0.08	0.65^∗∗∗^	–0.22^∗∗∗^	–							
5. Peer attachment (T1)	0.32^∗∗∗^	–0.12^∗∗^	0.23^∗∗∗^	–0.14^∗∗^	–						
6. Prosocial behavior (T1)	0.20^∗∗∗^	0.05	0.18^∗∗∗^	–0.06	0.40^∗∗∗^	–					
7. Aggression (T1)	–0.18^∗∗∗^	0.27^∗∗∗^	–0.17^∗∗∗^	0.28^∗∗∗^	–0.25^∗∗∗^	–0.27^∗∗∗^	–				
8. Prosocial behavior (T2)	0.21^∗∗∗^	–0.01	0.11^∗^	0.01	0.33^∗∗∗^	0.48^∗∗∗^	–0.27^∗∗∗^	–			
9. Aggression (T2)	–0.09	0.21^∗∗∗^	–0.15^∗∗^	0.24^∗∗∗^	–0.14^∗∗^	–0.16^∗∗∗^	0.48^∗∗∗^	–0.24^∗∗∗^	–		
10. Prosocial behavior (T3)	0.17^∗∗∗^	–0.01	0.19^∗∗∗^	0.02	0.25^∗∗∗^	0.45^∗∗∗^	–0.23^∗∗∗^	0.52^∗∗∗^	–0.21^∗∗∗^	–	
11. Aggression (T3)	–0.14^∗∗∗^	0.13^∗∗^	–0.19^∗∗∗^	0.11^∗∗^	–0.18^∗∗∗^	0.32^∗∗∗^	0.44^∗∗∗^	–0.28^∗∗∗^	0.52^∗∗∗^	–0.35^∗∗∗^	–

*T1, Wave 1; T2, Wave 2; T3, Wave 3. ^∗^*p* < 0.05; ^∗∗^*p* < 0.01; ^∗∗∗^*p* < 0.001.*

### Structural Equations Model

**FIGURE 1:**
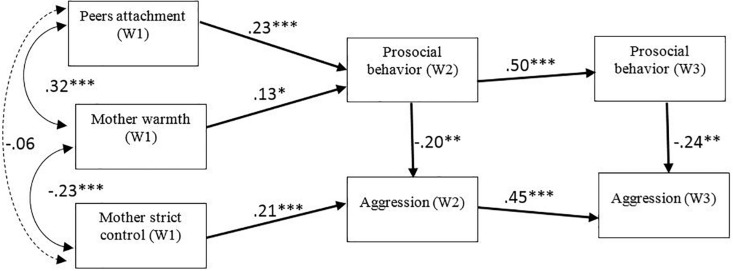
Longitudinal path model testing the associations between peer attachment (wave 1), mother warmth (wave 1), mother strict control (wave 1), aggression (wave 2, 3) and prosocial behavior (wave 2, 3). Gender was statistically controlled. Significant standardized coefficients are depicted. Indirect effects are depicted in bold. ^∗^*p* < 0.05; ^∗∗^*p* < 0.01; ^∗∗∗^ *p* < 0.001.

Two models were analyzed using structural equations modeling. The first model captured the relationships between the mother’s parenting styles (support and strict control) and prosocial behavior, and aggression, and between peer attachment and prosocial behavior, and aggression assessed in waves 2 and 3. In addition, the relationship between prosocial behavior and aggression was studied. Because the ANOVA had revealed statistically significant differences between genders, gender was used as a control variable of the mediators and outcomes in the models. These path coefficients are not depicted in the Figure 1.

The results indicate a good fit between the model and the empirical data: χ^2^ (4) = 29.03, *p* < 0.000. The following fit index was also obtained: CFI = 0.94. Finally, standardized root mean square residual was calculated: SRMR = 0.05. The model showed a very good fit. Values below 0.10 indicate acceptable error and values around 0.06 indicate a very good fit (Kline, 2011).

Bias corrected bootstrap confidence interval tests were conducted to examine indirect effects. Results showed that there was a significant indirect effect from peer attachment (T1) to prosocial behavior (T3) (β = 0.11; CI 95% = [0.03, 0.08]) via prosocial behavior (T2). There was also a significant indirect effect from peer attachment (T1) to aggression (T3) (β = −0.03; CI 95% = [−0.03, −0.005]), via prosocial behavior (T2 and T3), and via prosocial behavior (T2) and aggression (T2) (β = −0.02; CI 95% = [−0.02, −0.005]). There was also a significant indirect effect from parental support (T1) to prosocial behavior (β = 0.06; CI 95% = [0.01, 0.12]) at T3 via prosocial behavior (T2). Finally, there was a significant indirect effect from strict control (T1) to aggression (T3) (β = 0.09; CI 95% = [0.04, 0.15]) via aggression (T2).

**FIGURE 2:**
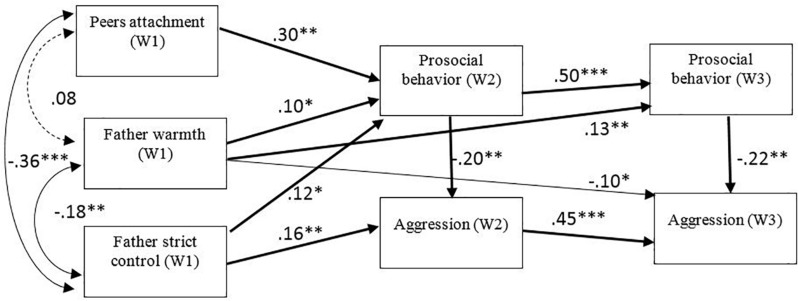
Longitudinal path model testing the associations between peer attachment (wave 1), father warmth (wave 1), father strict control (wave 1), aggression (wave 2, 3) and prosocial behavior (wave 2, 3). Gender was statistically controlled. Significant standardized coefficients are depicted. Indirect effects are depicted in bold. ^∗^*p* < 0.05; ^∗∗^*p* < 0.01; ^∗∗∗^*p* < 0.001.

The second model (Figure 2) captured the relationships between the father’s parenting styles (warmth and strict control) and prosocial behavior, and aggression, and between peer attachment and prosocial behavior, and aggression, in waves 2 and 3. In addition, the relationship between aggression and prosocial behavior was studied. In this analysis, gender was controlled. The results indicate a good fit between the model and the empirical data: χ^2^ (4) = 29.52, *p* < 0.000. The following fit indices were also obtained: CFI = 0.94 and SRMR = 0.05. Results of AIC and BIC showed that the first model had a better fit, with the lowest value in these indices.

Bias corrected bootstrap confidence interval tests were conducted to examine indirect effects. Results showed that there was a significant indirect effect from peer attachment (T1) to prosocial behavior (T3) (β = 0.15; CI 95% = [0.04, 0.11]) via prosocial behavior (T2). There was also a significant indirect effect from peer attachment (T1) to aggression (T3) (β = −0.03; CI 95% = [−0.03, −0.005]), via prosocial behavior (T2 and T3), and via prosocial behavior (T2) and aggression (T2) (β = −0.02; CI 95% = [−0.02, −0.005]). There was also a significant indirect effect from parental support (T1) to prosocial behavior (β = 0.05; CI 95% = [0.002, 0.06]) at T3 via prosocial behavior (T2). There was also a significant indirect effect from parental support (T1) aggression (β = −0.03; CI 95% = [−0.06, −0.001]) at T3 via prosocial behavior (T3). There was a significant indirect effect from strict control (T1) to prosocial behavior (T3) (β = 0.06; CI 95% = [0.002, 0.11]) via prosocial behavior (T2). Finally, there was a significant indirect effect from strict control (T1) to aggression (T3) (β = 0.07; CI 95% = [0.02, 0.12]) via aggression (T2).

## Discussion

The main aim of this longitudinal study was to determine the relationships among peer attachment, warmth from the mother and father, strict control by the mother and father, prosocial behavior, and aggression in adolescence.

With respect to the first hypothesis in which we argued that parenting styles built on warmth from the mother and father are related to greater peer attachment, whereas strict control by parents is not conducive to peer attachment, results in the concurrent model confirm that parenting characterized by affect and support from the mother is related to greater peer attachment, whereas strict control from the father does not promote peer attachment (Delgado et al., 2011; Samper et al., 2015). According to Attachment Theory (1988, 1995), the quality of the interactions with the main caregivers facilitates the development of the emotional regulation and well-being of the child, as well as the expectations that close relations will provide a safe haven and a secure base (Bowlby, 1988, 1995; Duarte-Rico et al., 2016). Studies have shown that the parenting style that best predicts peer attachment is support and control by the mother (Samper et al., 2015). In adolescents, a lack of support and control by the father predicts victimization in the youngest individuals, and strict control by the mother predicts victimization in the oldest individuals (Delgado et al., 2011).

On the other hand, we hypothesized a positive relationship between peer attachment and prosocial behavior and a negative relationship between peer attachment and physical and verbal aggression in adolescence. Results partially corroborate this hypothesis. Having one or more friends who provide support, security and trust in the early years of adolescence was found to relate to prosocial behavior 1 year later, and prosocial behavior in wave 2 was found to relate to subsequent prosocial behavior. Therefore, there was a positive relation between peer attachment and prosocial behavior in the longitudinal study (Van Hoorn et al., 2014). However, the relationship between peer attachment and prosocial behavior was observed in the first year, although this relationship was not observed in the next year. In fact, the best predictor of prosocial behavior (wave 3) was previous prosocial behavior (wave 2). Furthermore, there was a relation between peer attachment and aggression (wave 3) in the longitudinal model, but through prosocial behavior, in both wave 2 and wave 3, and through prosocial behavior and aggression, both variables of the second evaluation. Studies have shown that close relationships with peers can act as protective factors against aggression (Batanova and Loukas, 2014).

Regarding the relationship between the parenting styles of the father and mother and prosocial behavior and aggression at different moments during adolescence (Mestre et al., 2007; Richaud et al., 2013), the findings resemble those for the relationship between peer attachment and prosocial behavior. Adolescents that perceived support and communication from their father and mother behaved more prosocially 1 year later. Nonetheless, the best predictor of subsequent prosocial behavior was previous prosocial behavior. A direct relationship between parenting styles and prosocial behavior in wave 3 was observed only in the case of support from the father. Furthermore, a direct relationship between parenting styles and aggression in wave 3 was observed only in the case of support from the father. These results support socialization theories (Bandura, 1986; Hoffman, 2000), in which parents play a key role in promoting and fostering prosocial behavior. Surprisingly, there was a positive relation between father strict control and prosocial behavior. One reason to explain these results (Carlo et al., 2010b), could be that the measure used to evaluate parental strict control does not distinguish several types of parental strict control styles, such as psychological control and behavioral control, and it is possible that some types of control associate more to prosocial behavior than others. Additionally, these findings suggest that the effect of father strict control is ambiguous, on the one hand it encourages aggressivity, and on the other hand it also encourages prosocial behavior. Therefore, parental support is a more consistent predictor of prosocial behavior than parental strict control. Conversely, parenting styles characterized by strict control by the father and mother predicted greater physical and verbal aggression in adolescents. This relationship did not hold for subsequent aggression, although a strong correlation between aggression in waves 2 and 3 was observed.

As previously discussed, the influence of the mother’s warmth had a direct effect on the immediate behavior, whereas the perception of warmth from the father had an effect in subsequent years. Furthermore, the effect of strict control on aggressive behavior was similar for both parents. These findings highlight the importance of considering both the father and mother’s parenting styles when predicting aggression among adolescents (Hoeve et al., 2011). These findings also highlight the need for positive parenting styles characterized by support, affect and involvement from both parents in raising their children. Such parenting styles facilitate children socialization and prosocial development (Padilla-Walker et al., 2012; Scrimgeour et al., 2013). In contrast, paternal support was found to relate negatively to aggression during adolescence (Van der Graaff et al., 2012; Wahl and Metzner, 2012), whereas strict control had a positive association with aggression (Kuppens et al., 2013). Recently, in an attempt to break down gender stereotypes, men and women have questioned the traditional model (Heppner and Heppner, 2009). Nevertheless, in a recent study carried out by the Spanish Institute for Women’s Affairs (Instituto de la Mujer, 2013), the majority of Spanish women spend more time than men caring for their family.

Furthermore, prosocial behavior remained stable over the period under study, reflecting the findings of other studies examining this phenomenon in adolescents (Caprara et al., 2010; Carlo et al., 2010b). These findings could be due to social feedback processes, meaning, engagement in prosocial behavior earlier in life seems to promote later prosocial behavior (Carlo et al., 2010b). Conversely, physical and verbal aggression diminished progressively with each wave (Wahl and Metzner, 2012).

Gender differences were observed in prosocial behavior and aggression. Girls were more prosocial than boys throughout adolescence. In contrast, boys reported higher scores than girls in physical and verbal aggression. These findings are consistent with the view of women as being more empathetic and prosocial than men and the idea of greater aggression among adolescent boys (Mestre et al., 2009; Carlo et al., 2010a) but this finding should be taken with caution because it is based on self-reported behavior. These gender differences might be due to strong gender social roles that are established for men and women in many societies (Eagly, 2009). These gender-related stereotypes might inhibit prosocial behavior in adolescent boys and encourage prosocial behavior in adolescent girls. Girls reported greater peer attachment than boys of the same age (Delgado et al., 2011). In adolescent friendships with peers, greater empathy enables the child and adolescent to analyze, understand, and appreciate not only others’ behavior, but also the intentions, feelings, and reasons that motivate others. In this way, children and adolescents can understand that others’ intentions, feelings, and motives may differ from their own (Fuentes, 2005). This peer attachment correlated positively and significantly with prosocial behavior at the three moments evaluated, whereas the correlation between peer attachment and physical and verbal aggression was negative. These findings imply that prosocial behavior facilitates social relationships and that peer attachment facilitates prosocial behavior and curbs aggression (Samper et al., 2015). These results are consistent with the scholars that have asserted that peers could also serve as resources for fostering prosocial development (Van Hoorn et al., 2014). Regarding parenting styles, significant differences in the strict control of the mother were observed only as a function of gender. Boys perceived greater control from their mothers, whereas girls reported greater support from their fathers. The correlation analysis showed that parenting styles of the mother and father were related. Thus, adolescents perceived that their relationships with their mothers were similar to their relationships with their fathers (Tur-Porcar et al., 2004). Fathers are increasingly involved in their children’s education, acquiring an important parenting role (Ruiz-Hernández et al., 2018).

We can conclude that communication, warmth and positive control establish rules governing parent-child relationships (rules and compliance) that facilitate peer attachment (Herrero et al., 2006). In addition, this longitudinal study provides evidence of the importance of parenting styles in the prosocial development of adolescents, despite the increasingly prominent role of peers during adolescence. The findings also show that the parenting styles of the mother and father are related to their adolescent prosocial behavior (Padilla-Walker et al., 2012). In contrast, a lack of positive family relationship or an upbringing characterized by pathological control is related to aggressive behavior during adolescence (Van der Graaff et al., 2012; Batanova and Loukas, 2014).

Finally, results show the influence of the mother and father on prosocial and physical and verbal aggression during adolescence. In addition, strong peer attachment predicted prosocial behavior in subsequent years. Peer attachment had no direct effects on aggression during adolescence, although it did have an indirect effect through prosocial behavior, in the sense that peer attachment influenced prosocial behavior and this prosocial behavior curbed aggression (inverse correlation between prosocial behavior and aggression at different evaluations). Results therefore confirm that parenting styles and peer attachment relate to prosocial behavior and physical and verbal aggression. Hence, despite the growing presence of peers during adolescence, positive parenting styles remains important during this period (Delgado et al., 2011; Batanova and Loukas, 2014; Mestre, 2014; Samper et al., 2015). The Spanish National Youth Institute (INJUVE, 2012) reports that, for young Spaniards, family is the most important institution.

These findings show the role of parents and peers in adaptive, prosocial behavior that facilitates social relationships or, conversely, in maladaptive, aggressive behavior. Despite the increasingly prominent role of friends during the transition from childhood to adolescence, parents continue to play a key role in the personal and social development of adolescents. Therefore, programs aimed at preventing aggression should be designed considering the importance of stimulating and strengthening prosocial behavior, peer attachment and a family environment of affect, support and communication. Hence, preventative interventions should be promoted in family, school and peer environments. It is worth mentioning that the school is a particularly important stage for the study of socialization processes, as children and adolescents are with adults and their peers (Garaigordobil, 2014). The psycho-educational intervention programs whose aim is to prevent and reduce violent behavior among peers, should promote the improvement of the social climate, through communication, trust, and inclusion among peers, which in turn promotes the development of prosocial behavior.

The first limitation of this study is that it was based on adolescents self-report data. In future studies, parents, teachers and peers should be used as alternative information sources to provide data on prosocial behavior and aggression. Although the study was longitudinal, it focused on only a few years of adolescence. In future works we are thinking of using a multiple group SEM approach to examine whether child gender moderates the pattern of relations across the two models as well as include other demographic factors such as income and parental education. Despite these limitations, we feel that the findings of this study provide novel comparative information regarding the importance of parenting styles and peer attachment in preventing aggression throughout adolescence and the importance of prosocial behavior as an alternative to aggression.

## Data Availability Statement

All datasets generated for this study are included in the manuscript/supplementary files.

## Ethics Statement

The participation of the adolescents was voluntary and anonymous, taking into consideration all ethical principles pertaining to research with human beings included in the Helsinki Declaration, under the current regulations. The research project had a favorable response from the university ethics committee because it is required for the concession of these grants GVPROMETEO/2015/003, PSI2016-78242-R-AEI/FEDER, and AICO/2016/090.

## Author Contributions

EM, AL, and BM: materials and method, results, and references. PS and MM: introduction and discussion.

## Conflict of Interest

The authors declare that the research was conducted in the absence of any commercial or financial relationships that could be construed as a potential conflict of interest.

The reviewer AM declared a shared affiliation, with no collaboration, with several of the authors, EM, AL, PS, MM, to the handling Editor at the time of review.
